# Crystal Structure of the Mineralocorticoid Receptor DNA Binding Domain in Complex with DNA

**DOI:** 10.1371/journal.pone.0107000

**Published:** 2014-09-04

**Authors:** William H. Hudson, Christine Youn, Eric A. Ortlund

**Affiliations:** 1 Department of Biochemistry, Emory University School of Medicine, Atlanta, Georgia, United States of America; 2 Discovery and Developmental Therapeutics, Winship Cancer Institute, Emory University School of Medicine, Atlanta, Georgia, United States of America; University of Ulm, Germany

## Abstract

The steroid hormone receptors regulate important physiological functions such as reproduction, metabolism, immunity, and electrolyte balance. Mutations within steroid receptors result in endocrine disorders and can often drive cancer formation and progression. Despite the conserved three-dimensional structure shared among members of the steroid receptor family and their overlapping DNA binding preference, activation of individual steroid receptors drive unique effects on gene expression. Here, we present the first structure of the human mineralocorticoid receptor DNA binding domain, in complex with a canonical DNA response element. The overall structure is similar to the glucocorticoid receptor DNA binding domain, but small changes in the mode of DNA binding and lever arm conformation may begin to explain the differential effects on gene regulation by the mineralocorticoid and glucocorticoid receptors. In addition, we explore the structural effects of mineralocorticoid receptor DNA binding domain mutations found in type I pseudohypoaldosteronism and multiple types of cancer.

## Introduction

Steroid hormones are powerful regulators of homeostatic functions such as cell growth, immunity, reproduction, and metabolism [1]. Steroid hormones exert their effects by binding to steroid hormone receptors (SRs), which include the estrogen receptors as well as members of the NR3C subfamily (i.e. the mineralocorticoid, glucocorticoid, androgen, and progesterone receptors). Upon ligand binding, SRs translocate from the cytoplasm, where they are bound to heat shock proteins, to the nucleus where they bind their DNA response elements and regulate the transcription of hundreds of genes [2]. The potent transcriptional activity of SRs combined with a very high affinity for their endogenous ligands allows small concentrations of steroid hormones to coordinate diverse cellular processes.

Protein domain structure is conserved throughout the NR3C family. The SRs contain a N-terminal transactivation domain of variable length, a DNA binding domain (DBD) containing two Cys_4_ zinc fingers, and a flexible hinge connecting the DBD to the ligand binding domain (LBD) [3]. The N-terminal transactivation domain and the hinge are of variable lengths and not well conserved among the NR3C receptors. While hormone preference differs among NR3C receptors due to sequence differences in the LBD, the DBDs are highly conserved, conferring overlapping DNA binding preferences for all members of this subfamily. However, the mineralocorticoid receptor (MR) and glucocorticoid receptor (GR), which diverged after a gene duplication of an ancient corticoid receptor, also show overlap in hormone preference [4]. MR responds to aldosterone, 11-deoxycorticosterone and cortisol, while GR is selective for cortisol only.

Expression of MR is tissue-specific, with highest concentrations found in the kidney, brain, and heart [5]–[8]. MR is involved in responses to stress and is basally activated in the brain [9], [10], but is most commonly studied for its role in vascular health and salt and water balance. MR knockout mice develop normally, but die near postnatal day 10 from renal sodium and water loss [11]. These mice exhibit extreme hyperactivation of the renin-angiotensin system, with elevated renin, angiotensin II, and aldosterone levels [11]. Aldosterone promotes atherosclerotic plaque formation [12], and its levels serve as a predictor of acute ischemic events and death in patients with coronary artery disease [13]. These findings have led to the use of MR antagonists to treat heart failure [14].

Both MR and GR bind glucocorticoid response elements (GREs) on genomic DNA to control target gene expression [15], [16]. Administration of glucocorticoids prolongs the survival of MR^−/−^ mice, suggesting overlapping - but not fully compensatory - functions of GR and MR [11]. Additionally, GR and MR differentially regulate cellular functions such as inflammation, with MR often acting as a pro-inflammatory factor and GR acting as an anti-inflammatory factor [17]–[20]. These differences may be due to opposing effects on gene regulation. For example, MR upregulates the expression of the pro-inflammatory gene *ICAM1* [21], while GR acts to transrepress its expression [22].

The mechanisms that underlie such differential gene regulation by receptors with similar sequence and overlapping preferences for ligand and DNA binding are unknown. In this report, we determine the first crystal structure of the MR DBD in order to provide a framework for elucidating the subtle differences between the corticosteroid receptors and interpreting the biology of disease-associated mutations.

## Materials and Methods

### Protein expression and purification

The DNA binding domain (DBD) of the human MR (amino acids 593–671, UniProt P08235.1) was cloned into the pMCSG7 vector, which contains a 6X-histidine tag. Both the MR and GR DBDs were expressed and purified as described previously [23]. Briefly, BL-21 (DE3) pLysS *E. coli* transformed with the expression construct were grown in TB media. At an OD_600_ of ∼0.8, cultures were induced with 300 µM IPTG for four hours at 30°C. Cells were pelleted via centrifugation at 4,000 *g* for 20 minutes and frozen at −80°C until purification.

For purification, cells were thawed and resuspended in a buffer containing 1 M NaCl, 20 mM Tris-HCl pH 7.4, 5% glycerol, and 25 mM imidazole. Cells were lysed on ice via sonication and centrifuged for 1 hour at 4°C and 35,000 *g*. DBD was purified from the supernatant using a 5 mL HisTrap affinity column followed by gel filtration with a HiPrep 26/60 S300 Sephacryl column (GE Healthcare) into a buffer containing 100 mM NaCl, 20 mM Tris-HCl pH 7.4, and 5% glycerol. Protein was concentrated to 3 mg/ml, flash frozen, and stored at −80°C.

### Crystallization, data collection, and structure determination

Crystals of the MR DBD – GRE complex were grown by hanging-drop vapor diffusion in 0.2 M sodium malonate and 12% PEG 3350 at a protein concentration of 3.0 mg/ml and a 2∶1 molar ratio of DNA to protein. Crystals were cryoprotected in 0.2 M sodium malonate, 12% PEG 3350 and 20% glycerol and flash cooled in liquid N_2_.

Data were collected remotely on the 22-ID beamline at the Southeast Regional Collaborative Access Team (SER-CAT) at the Advanced Photon Source (Argonne, IL, USA). Data were processed using HKL-2000 software and phased using previously solved structures of the GR DBD bound to GREs [24], [25]. Phasing, refinement, and omit map generation were performed in the PHENIX software suite (version 1.9_1692) [26]. Model building was performed in COOT (version 0.6.1) [27]. The data are highly anisotropic and completeness is only 67% in the highest resolution shell (2.39–2.30 Å; Table 1) despite 49.2% of reflections in the shell having an *I/*σ*I* >5. To balance completeness and to avoid throwing out useful data available data, 2.39 Å was chosen as the resolution cutoff for refinement. The PyMOL software suite (Schrödinger, LLC) was used to visualize the structure and generate figures. Amino acids are numbered according to the human MR sequence (UniProt P08235.1). 3DNA was used to analyze nucleic acid structure [28], and the PISA server was used to calculate buried surface areas of each interface [29]. The coordinates and structure factors for the MR DBD – GRE complex were deposited in the Protein Data Bank under accession code 4TNT.

**Table 1:** Data collection and refinement statistics.

	MR DBD - GRE
**Data collection**	
Space group	C222_1_
Cell dimensions	
* a*, *b*, *c* (Å)	74.1, 115.1, 81.4
* Α, β, γ* (°)	90.0, 90.0, 90.0
Resolution (Å)	2.39 (2.48–2.39)*
*R*_merge_	7.8 (45.4)
*I/*σ*I*	25.3 (2.4)
Completeness (%)	92.7 (66.7)**
Redundancy	4.1 (3.0)
**Refinement**	
Resolution (Å)	2.39
No. reflections	13291
*R*_work_/*R*_free_	21.9/25.8
No. atoms	
Protein	1084
DNA	691
Water	4
*B*-factors	
Protein	71.0
DNA	86.0
Water	60.1
R.m.s. deviations	
Bond lengths (Å)	0.010
Bond angles (°)	1.23

*Data for highest resolution shell are in parentheses.

**Data are 94.1% complete to 2.59 Å. Data were collected from a single crystal. The estimated isotropic and anisotropic Wilson B for the data is 52.94 and 86.19 Å^2^, respectively.

### Nucleic acid binding assay

A synthesized 6-carboxyfluorescein (6-FAM) labeled GRE (Integrated DNA Technologies) was annealed in 10 mM NaCl and 20 mM Tris-HCl pH 8.0 by heating to 90°C in a 1 L water bath and slow cooling to room temperature. The GRE sequences used for binding were 5′-[FAM]CCAGAACAGAGTGTTCTGA-3′ and 5′-TCAGAACACTCTGTTCTGG-3′, where [FAM] indicates the position of 6-FAM. Indicated amounts of DBD were added to wells containing 10 nM of 6-FAM–labeled GRE, and formation of DBD-GRE complexes was monitored by fluorescence polarization with a Biotek Synergy plate reader at an excitation wavelength of 485 nm and emission wavelength of 528 nm. Reactions were performed in buffer containing 100 mM NaCl, 20 mM Tris-HCl (pH 7.4), and 5% glycerol. Prism version 6.0 d (Graphpad Software, Inc.) was used for data analysis and graph generation.

### Cancer mutations

Cancer mutations were accessed via the cBioPortal for Cancer Genomics [30].

### Sequences

Sequence numbering for the SRs are for the human proteins and derived from the following sequences: androgen receptor, UniProtKB P10275.2; progesterone receptor, UniProtKB P06401.4; mineralocorticoid receptor, UniProt P08235.1; glucocorticoid receptor, GenBank ADP91252.1.

## Results

### Crystal structure of the MR DBD – GRE complex

**Figure 1:**
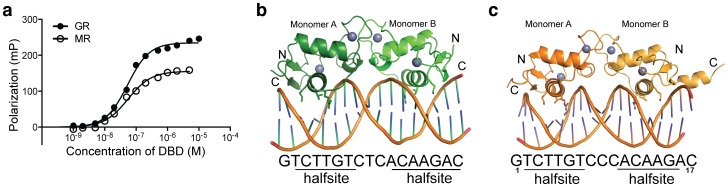
Structure of the human mineralocorticoid DNA binding domain in complex with a glucocorticoid response element. (a) The MR DBD binds to a GRE with approximately the same affinity as the GR DBD. (b) Overall structure of the MR DBD (green) bound to a 17 base pair GRE. The sequence of the element, along with the two bound half sites, is shown below the structure. In both panels (b) and (c), the structure shown depicts the asymmetric unit of the crystal structure and separate GR monomers are differentially colored. (c) Structure of the GR DBD (orange) bound to a similar GRE, with sequence and half sites indicated below. Panel (c) is derived from the structure of the GR DBD bound to the FKBP5 GRE, PDB 3G6P [24].

To ensure proper folding and activity, we tested the ability of purified MR DBD and GR DBD to bind to a fluorescently labeled GRE via fluorescence polarization (Figure 1a). Both proteins showed similar affinity for the element, at 55 nM and 53 nM for MR and GR, respectively. These are consistent with previous reports of MR – DNA binding on the order of 10 nM [31]. Both proteins showed similar, slight positive cooperativity in DNA binding, which would likely be enhanced by lower salt concentrations [32].

We then crystallized the MR – GRE complex, obtaining small crystals that anisotropically diffracted to 2.4 Å (Table 1). The crystal structure of the MR – GRE complex reveals a canonical SR DBD dimer bound to the GRE sequence via interaction two DNA half sites (Figure 1b). As expected, the structure is very similar to structure of GR DBD – GRE complexes (r.m.s.d. <1.0 Å; Figure 1c). As multiple GR – GRE complexes have previously been solved, we compared our novel MR-GRE structure to PDB 3G6P, which contains GR in complex with a GRE derived from the FKBP5 promoter [24]. This GRE is nearly identical to the sequence contained in the crystal structure reported here (Figure 1b,c). One GR DBD monomer (monomer B in Figure 1c) contains a C-terminal α-helix when bound to the FKBP5 GRE (Figure 1c); the MR DBD structure reported here does not exhibit such a structure. However, not all GR DBDs form this helix when bound to DNA, including monomer A in the GR DBD – FKBP5 GRE structure (Figure 1c).

### Sequence-specific contacts between MR and GREs

**Figure 2:**
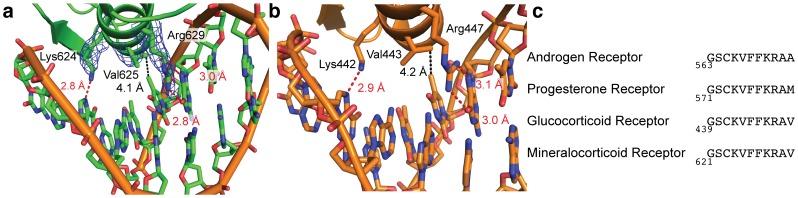
Sequence-specific DNA recognition by the MR DBD. (a) Three residues mediate sequence-specific contacts by the MR DBD DNA reading helix. Lysine 624 makes a hydrogen bond with a guanine base, valine 625 makes van der Waals contacts with a thymine base, and arginine 629 makes two interactions with a guanine base. Electron density (composite omit 2F_o_−F_c_ map with simulated annealing, contoured to 1 σ) is shown for the three protein side chains. (b) GR recognizes GREs in an identical manner as the MR DBD, using lysine 442, valine 443, and arginine 447 to contact analogous bases. (c) Sequence alignment showing conservation of the DNA reading helix among the NR3C receptors.

Inspection of the MR – DNA interface reveals three amino acids that make sequence-specific contacts with GRE bases (Figure 2a). The terminal nitrogen of lysine 624 forms a hydrogen bond with the N7 position of guanine 3. On the opposite DNA strand, valine 25 makes van der Waals contacts with C7 of thymine 13, and arginine 629 makes two interactions with guanine 12 at the O6 and N7 positions. These interactions are supported by excellent electron density (Figure 2a). These interactions are conserved in the glucocorticoid receptor, which contacts DNA in an identical fashion (Figure 2b). Lysine 442, valine 443, and arginine 447 in GR make contacts with a guanine, thymine, and guanine, respectively. These DNA-reading amino acids are strictly conserved in the four steroid receptors in the GR/MR-like subfamily (Figure 2c), and their mutation in GR leads to deficiencies in both DNA and RNA binding in GR [33], [34].

### Lever arm conformation of MR

**Figure 3:**
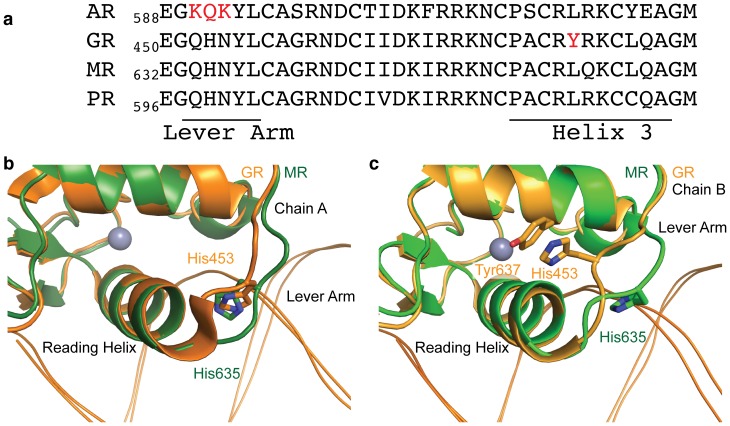
Lever arm conformation differs between MR and GR. (a) Sequence alignment of the lever arm through helix 3 of the NR3C receptors. AR and GR contain divergent sequence at the lever arm and helix 3, respectively (red). In a dimer of GR molecules on DNA (see Figure 1c), the side chain of histidine 453 can assume two conformations. (b) Monomer A of the GR – FKBP5 GRE complex contains histidine 453 in a “flipped” conformation, where the histidine side chain sits between the DNA and DBD reading helix; a similar conformation is seen in monomer A of the MR DBD – GRE complex. (c) However, histidine 453 in the second GR DBD monomer assumes a “packed” position against tyrosine 637 in the core of the GR DBD fold. This conformation does not occur in the MR DBD – GRE structure, likely due to the presence of a leucine rather than tyrosine at position 660 (GR position 478). In panels (b) and (c), DNA is shown as a ribbon helix below the protein.

Previous studies have proposed that DNA sequence allosterically modulates GR’s structure, in turn affecting transcriptional activation [24], [35]. One possible mechanism for such allosteric modulation may be structural changes in the “lever arm” of steroid receptors, which connects the DNA reading helix of the receptor to its dimerization loop (Figure 3a). Mutation of lever arm residues affects transcriptional activation [24], and one GR splice variant, GRγ, contains a single arginine insertion into the lever arm. This insertion has the ability to affect both GR’s binding to target DNA as well as its transcriptional activity [36].

The lever arm sequence of MR is identical to that of GR and the progesterone receptor (PR), although the androgen receptor (AR) contains three amino acid changes in this region (Figure 3a). A key structural element of the lever arm is the position of histidine 453 in GR, which is also strictly conserved in MR (histidine 635). In GR, the side chain of histidine 453 can assume a “flipped” conformation, where it occupies a position between the DNA and the reading helix (Figure 3b). This conformation can also be seen, with minor variations, in the crystal structure of MR bound to a GRE (Figure 3b). However, histidine 453 can also assume a “packed” conformation in GR, wherein the side chain rests between GR helices and stacks against a tyrosine residue in helix 3 of the DBD fold (Figure 3c). This tyrosine, residue 478, is unique to GR and is conserved as a leucine in the other NR3C family receptors (Figure 3a). This amino acid difference likely reduces the stability of the “packed” conformation, explaining why this conformation is not observed in the MR-GRE structure (Figure 3c). This single amino acid change may cause MR to respond differently than GR to identical sequence elements and alter any potential protein-DNA mediated allostery. However, conclusions regarding the structure and function of the lever arm may be confounded by crystal packing contacts found in the lever arm in many GR DBD-DNA structures as well as in chain B (but not chain A) of the structure reported here.

### Dissecting the protein-DNA interface

**Figure 4:**
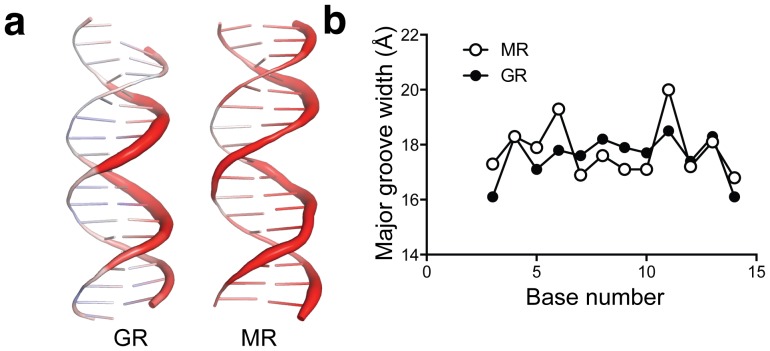
Analysis of the MR – DNA interface. (a) Thermal motion of GREs when bound to GR (left) and MR (right). Thicker, red sections of DNA indicate higher B-factors and therefore higher thermal motion. (b) Major groove width at each position of the GRE when bound to the MR and GR DBDs. (c).

Recent studies have shown that the shape of DNA regulates protein-DNA binding whereby occupancy of one protein binding site on a DNA double helix affects the occupancy of additional binding sites in a periodic manner [37]. This phenomenon may be exploited by the GR to prevent cooperative dimerization at negative glucocorticoid response elements (nGREs) [23]. The MR – GRE structure reveals that the MR dimer perturbs DNA in a very similar manner as a GR dimer (Figure 4a). Both receptors induced a similar widening of the DNA major groove to 18 Å (Figure 4b). This is noticeably distinct from interactions of GR with nGREs, where the major groove is constricted relative to GREs bound to GR [23].

We also analyzed the buried surface area of each MR monomer-DNA interface as well as the MR dimer interface. The MR dimer interface buries 574 Å^2^ solvent accessible surface, similar to the GR dimer interface when bound to the FKBP5 GRE, which buries 555 Å^2^. However, each MR – GRE interface is comprised of a much smaller surface area than the corresponding GR – GRE interface. The two MR DBD – DNA interfaces bury 373 Å^2^, and 369 Å^2^, compared to 554 Å^2^ and 520 Å^2^ for GR. This is consistent with GR’s potential ability to bind to DNA as a monomer [23].

## Discussion

**Figure 5:**
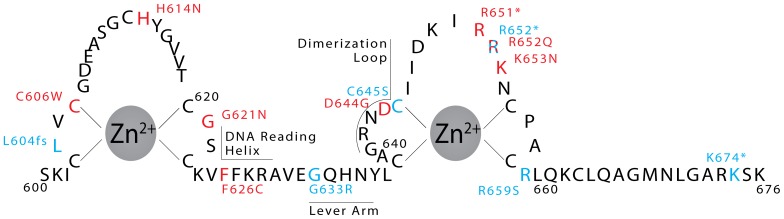
MR DBD mutations found in disease. Mutations found in type I pseudohypoaldosteronism are in blue and mutations found in cancer are in red. An asterisk indicates a nonsense mutation, and fs indicates a frameshift mutation.

**Figure 6:**
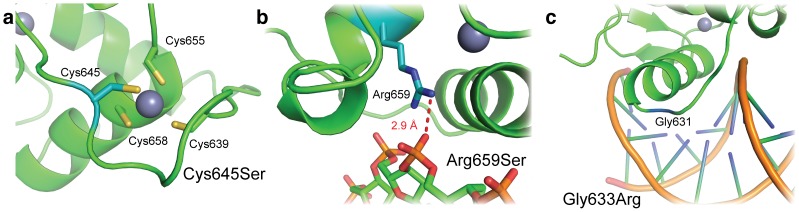
MR DBD mutations driving PHA1. Mutated residues are shown in blue. (a) Cysteine 645 is one of four cysteines that coordinate a Zn^2+^ ion in MR’s second zinc finger. Its mutation to serine would destroy the zinc finger fold of the DBD. (b) Arginine 659 makes non-specific interactions with the DNA backbone, and is mutated to serine in some cases of PHA1. (c) Glycine 633 is part of the DBD lever arm, which is important for receptor activation [24]. Mutation of this residue to arginine affects receptor activation without affecting its affinity for DNA [39].

Mutations within MR are the primary cause of type 1 pseudohypoaldosteronism, or PHA1 [38]. Many of these mutations target the receptor DBD, including nonsense, missense, and frameshift mutations (Fig. 5). The missense mutations in PHA1 include the mutation of the Zn^2+^-coordinating cysteine 645 to serine, which would be devastating for folding of the zinc finger (Figure 6a). Additional PHA1 missense mutations include the mutation of lever arm glycine 633 to arginine [39] and the mutation of arginine 659 to serine at the DNA binding interface [38]. The arginine 659 mutation removes a charge-charge interaction between the MR DBD and the DNA backbone, likely reducing DNA binding activity without altering sequence specificity (Figure 6b). The lever arm mutation of glycine 633 to arginine does not alter DNA binding affinity [39], and the structure of the MR DBD reveals this residue is solvent exposed (Figure 6c). However, this mutation reduces MR’s transactivation ability by 40% compared to wild-type receptor, supporting the lever arm’s predicted role in receptor activation [24].

In addition to endocrine disorders, steroid receptors frequently assume malicious roles in cancer, with PR and the estrogen receptor (ER) often driving breast cancer growth and AR driving prostate cancer cell growth [40]–[42]. While MR and GR are less studied with respect to their action in cancer cells, there is accumulating evidence that these receptors also play key roles in neoplastic diseases [43]–[46]. A recent study demonstrated that a decrease in MR expression was associated with increased angiogenesis and poor patient survival in colorectal cancer [45]. MR is mutated in up to 6% of colorectal cancer samples on the cBioPortal database and is also frequently (≥5%) altered in skin cutaneous melanoma, uterine, bladder, and stomach cancers [30], [47].

**Figure 7:**
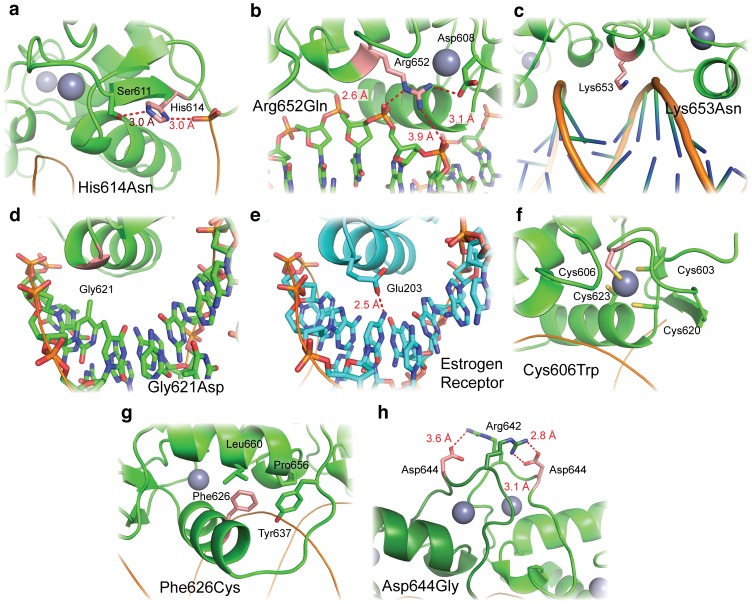
MR DBD mutations found in cancer. Mutated residues are shown in red. (a) Histidine 614 interacts with both the DNA backbone and serine 611. (b) Similarly, arginine 652 also interacts with the DNA backbone and a neighboring amino acid, aspartic acid 608. (c) Lysine 653 is within an appropriate distance to make non-specific contacts with the minor groove of a GRE. (d) Glycine 621 is mutated to aspartic acid in a stomach cancer sample. In the estrogen receptor, the homologous amino acid is the similar glutamic acid, which participates in base-specific DNA recognition (panel e). (f) Cysteine 606 is one of four cysteine residues to coordinate a Zn^2+^ ion in one of MR’s two zinc fingers. (g) Phenylalanine 626 comprises part of the hydrophobic core of the DBD. (h) Aspartic acid 644 is a key mediator of MR dimerization, forming a salt bridge with arginine 642 of the second monomer.

Several mutations found in cancer affect the MR DBD, including nonsense mutations that truncate part of or the entire domain (Figure 5). Four missense mutations affect the DNA binding interface of the MR DBD (Figure 7a–d). Histidine 614, which interacts with both the phosphate DNA backbone and a serine side chain, is mutated in a kidney renal papillary cell carcinoma sample to asparagine (Figure 7a). Arginine 652, which also interacts with the DNA backbone, is mutated to glutamine in a uterine corpus endometrioid carcinoma sample (Figure 7b). MR Lysine 653 is mutated to asparagine in multiple cancer types. This residue may make non-specific contacts with the minor groove, but does not have strong electron density to support its side chain position (Figure 7c). The most interesting mutation at the DNA interface is that of glycine 621 to aspartic acid (Figure 7d). This glycine residue is strictly conserved in GR, AR, and PR, but ER contains a glutamic acid at the homologous position (Figure 7e) [48]. Mutation of the homologous residue in GR, glycine 439, to aspartic acid results in a DNA binding domain that poorly discriminates among GREs and estrogen response elements [49].

Two additional cancer mutations target the hydrophobic core of the MR DBD (Figure 7f, g). One mutation found in a glioblastoma multiforme patient targets cysteine 606, which is one of four cysteine residues that coordinate a Zn^2+^ ion in one of MR’s two zinc fingers (Figure 7f). Like the mutation of cysteine 656 in PHA1, this mutation to tryptophan would be devastating for folding of the DBD. A second cancer mutation within the hydrophobic core, phenylalanine 626 to cysteine, may also affect the DBD’s core fold (Figure 7g). Finally, one interesting mutation in colorectal cancer targets the dimerization interface of the MR DBD (Figure 7h). In this case, aspartic acid 644 is mutated to glycine; this aspartic acid participates in two salt bridge interactions that link the two MR DBD monomers. Such a mutation may affect cooperative binding of the receptor to DNA and subsequent gene activation.

In addition to PHA1 and cancer, MR mutation also occurs in hypertension [50]; mutations of genes in steroid metabolic pathways upstream of MR can lead to similar disorders [51]. Some MR DBD mutations in PHA1 are frameshift or nonsense mutations [38], but many are missense mutations that affect the dimerization, DNA binding, or hydrophobic core structure of the domain (Figure 6). The MR mutations found in cancer are very similar, affecting the fold of the DBD, its DNA binding interface, and its dimerization loop. Since these types of mutation diminish MR’s transcriptional activity in PHA1, the MR mutations found in cancer also likely abrogate receptor activity. This is consistent with the decreased MR expression found in some types of cancer [52], [53].

Such mutations found in PHA1 and cancer may also lead to structural changes of elements flanking the DBD, such as the nuclear localization sequences immediately to the C- and N-terminus of the MR DBD [54]. Several post-translational modifications also occur at the DBD flanks, including acetylation at Lys677 [55] and phosphorylation at Ser601 [54]. Amino acid changes in MR are not limited to cancer and PHA1: numerous human SNPs within MR’s coding region have been identified, including the change of valine 617 to alanine in the DBD (rs373194830) [56]. This mutation likely has a minimal effect on MR activity, since the androgen receptor contains an alanine at the homologous position. However, it is possible that such SNPs lead to quantifiable physiological differences, as has been noted with MR polymorphisms in the N-terminal domain [9]. Finally, one MR mutation in cancer may change the DNA binding specificity of the receptor. The stomach cancer mutation of glycine 621 to aspartic acid mirrors the mutation of glycine 439 previously performed with the GR [49]. This mutation within GR led to a DBD that poorly discriminated among estrogen and glucocorticoid response elements [49]. Such diverse DNA recognition *in vivo* may allow for the receptor to activate a more diverse set of target genes than wild type MR.

MR and GR can heterodimerize [57], implying that MR mutations that affect the core structure and dimerization of the receptor may also affect responses to glucocorticoids. Other domains of MR are key for other protein-protein interactions, including the unstructured N-terminal domain [58]. Common polymorphisms in the N-terminal domain lead to phenotypic changes in stress response, including altered saliva production and heart rate [9].

In addition to its relevance to human disease, the comparison between MR and GR is interesting due to their differential ability to modulate gene expression despite sharing overlapping DNA and ligand binding properties. The DBDs of GR and MR make identical contacts with DNA, but GR buries a larger surface area at the GRE interface and makes very favorable monomer-DNA interactions at nGREs [23]. Although MR binding to consensus nGREs [59] has not been tested, this difference in DNA binding may allow GR to bind to a greater diversity of DNA sequences. ChIP-seq analyses have found a large number of motifs at GR binding sites on genomic DNA, including not only the GRE but also AP-1, AML1, UNKN, NF-κB, HNF3, TAL1, and NF1 response elements [60]. In ChIP-seq studies of the MR, only palindromic motifs were explored; it is unclear whether MR binds a similarly wide array of genomic binding sites [15]. Future work is required to determine how such similar receptors can effect disparate function *in vivo* and whether this disparate function is based on differences in DNA binding preference.
